# Apatite Formation: Why It May Not Work as Planned, and How to Conclusively Identify Apatite Compounds

**DOI:** 10.1155/2013/490946

**Published:** 2013-07-29

**Authors:** Christophe Drouet

**Affiliations:** CIRIMAT Carnot Institute-UMR CNRS/INPT/UPS 5085, University of Toulouse, Ensiacet, 4 Allée Emile Monso, 31030 Toulouse Cedex 4, France

## Abstract

Calcium phosphate apatites are inorganic compounds encountered in many different mineralized tissues. Bone mineral, for example, is constituted of nanocrystalline nonstoichiometric apatite, and the production of “analogs” through a variety of methods is frequently reported. In another context, the ability of solid surfaces to favor the nucleation and growth of “bone-like” apatite upon immersion in supersaturated fluids such as SFB is commonly used as one evaluation index of the “bioactivity” of such surfaces. Yet, the compounds or deposits obtained are not always thoroughly characterized, and their apatitic nature is sometimes not firmly assessed by appropriate physicochemical analyses. Of particular importance are the “actual” conditions in which the precipitation takes place. The precipitation of a white solid does not automatically indicate the formation of a “bone-like carbonate apatite layer” as is sometimes too hastily concluded: “all that glitters is not gold.” The identification of an apatite phase should be carefully demonstrated by appropriate characterization, preferably using complementary techniques. This review considers the fundamentals of calcium phosphate apatite characterization discussing several techniques: electron microscopy/EDX, XRD, FTIR/Raman spectroscopies, chemical analyses, and solid state NMR. It also underlines frequent problems that should be kept in mind when making “bone-like apatites.”

## 1. Introduction

Synthetic-calcium-phosphate-apatite-based compounds are frequently encountered in the literature dealing with bone tissue engineering. This fact is not surprising though, given the apatitic nature of bone mineral itself [1]. Detailed investigations on this biomineral (e.g., [2–8]) have revealed its nanocrystalline, nonstoichiometric, and hydrated characters, as well as specific surface features involving labile ions in nonapatitic chemical environments, potentially exchangeable with ions contained in surrounding fluids. Similar characteristics were also pointed out for synthetic biomimetic analogs (e.g., [9]), allowing one to prepare bioactive ceramics [10]. 

Such biomimetic nanocrystalline apatites exhibit an overall chemical composition that can generally be described by formulas of the type Ca_10−*x*_(PO_4_)_6−*x*_(HPO_4_)_*x*_(OH)_2−*x*_ [11] or Ca_10−*x*−*Z*_(PO_4_)_6−*x*_(HPO_4_)_*x*_(OH)_2−*x*−2*Z*_ [12], where *x* and *Z* depend on conditions of formation and state of ageing (maturation). It should be stressed here that the physico-chemical characteristics of nanocrystalline apatites—whether of biological or synthetic origin—differ significantly from those of stoichiometric hydroxyapatite (HA), Ca_10_(PO_4_)_6_(OH)_2_, especially in terms of stoichiometry, crystal size, crystal disorder, unit cell parameters, surface features, and hydration state [13].

While the synthesis of apatite materials for the preparation of implantable bioceramics is bound to occupy a privileged place in bone applications thanks to (sub)structural and compositional features close to those of bone mineral, it is not suited—in a self-supported way—for load-bearing applications that require stronger mechanical properties. Metal implants then appear as the most commonly used candidates (e.g., hip or knee prostheses), despite potential drawbacks such as poor bone-bonding abilities or allergy issues [14]. In this case, the chemical quality of the interface between the implant and the surrounding bone tissue is of prime importance for the success and durability of implantation [15]. This point has led many researchers and industrial companies to investigate and develop deposition processes of apatite compounds on metal implants (e.g., by plasma spray technologies) in view of favoring bone-bonding properties [16–18]. Also, more generally, tests are often carried out in supersaturated solutions so as to evaluate the ability of a surface to induce the nucleation and growth of a “bone-like” apatite layer [19]. In these cases, the term “supersaturated” implicitly refers to hydroxyapatite (solubility product *pK*_sp_ (25°C) = 117 [20] for the formula Ca_10_(PO_4_)_6_(OH)_2_). Several types of supersaturated solutions have been used, the most famous probably being the so-called SBF solution (Simulated Body Fluid) [19, 21] or its multiples (e.g., SBF × 1.5) [22, 23]. Tests are generally performed for several days and often at 37°C to approach physiological conditions. Although care should be taken when drawing conclusive statements on a material “bioactivity” on the sole basis of such laboratory tests (run out of the body and in the absence of cells or often of proteins), such assays may however be informative, for example, for a preliminary “ranking” of implants capabilities [21], but it has to be kept in mind that additional data are needed (e.g., cell behavior assessments and then *in vivo* confirmations) to draw a more accurate picture of the actual bioactivity or expected biological behavior of the samples tested.

In all the preceding statements, the formation of an apatite phase is a specific concern, whether for the production of self-supported bioactive ceramics, for the setup of a coating on implants for improving bone-bonding abilities, or else for drawing relative “bioactivity” information. A great deal of papers and congress communications refer to these aspects, and conclusive statements are commonly drawn. Unfortunately, adequate physico-chemical characterization data are not always attached to such statements, and the establishment of the “apatitic” nature of samples or deposits is sometimes unconvincing or even dubious. The announced conditions of preparation of a compound considered to be apatite sometimes depart from the conventional conditions of stability of apatite, requiring characterization proofs. In other instances, the sole observation of a white deposit after immersion in supersaturated media is considered as a solid proof for concluding that a “bone-like carbonated apatite layer” has been formed, which is obviously not scientifically satisfactory.

Various aspects should in contrast be taken into account before making conclusive statements. The chemistry of calcium phosphates is indeed very rich, with numerous possible phases (beside apatite) able to form depending on experimental conditions [24]. Also, the “real” conditions of treatments are sometimes slightly different from the “intended” ones, due, for example, to experimental mistakes or approximations, and some steps in the synthesis/coating process may not be adequately performed. Furthermore, the substrate itself might be able to react with the immersion solution, for instance, by releasing/capturing some ions or molecular entities into/from the surrounding medium, thus modifying its actual surface state or the external conditions (e.g., effect on the pH of the medium). Finally, inadequate conditions may lead to variations in supersaturation of pH along the process.

All of these considerations point to a situation that is more complex than may be initially thought. All in all, the conclusion that an *apatite* phase has been obtained requires, as in every reliable materials science study, adequate physico-chemical characterization. This contribution is intended to review the main characteristics to be expected from a calcium phosphate apatite compound, drawn from complementary characterization techniques. The cases of hydroxyapatite and of a nanocrystalline apatite of rather low maturation state are examined for establishing typical “identity cards” for such materials. Characteristics of other minerals that may possibly also be encountered in the above-mentioned contexts are also reviewed, and some frequent issues are commented upon. 

## 2. Results and Discussion

### 2.1. Apatite Formation: Why It May Not Work as Planned…

In this first section, the idea is to provide readers with elements pointing out that apatite formation is not a trivial phenomenon. Whether due to inexact concentration calculations, unexpected alteration/equilibration of a substrate surface upon immersion, or else because of incomplete processing steps, several factors could in fact lead to other outcomes than planned. The following paragraphs describe some of the most likely causes of experimental failure or mistakes to avoid.

#### 2.1.1. The Impact of Incomplete Washing

Most laboratory tests aiming at evaluating the ability of solid surfaces to get covered by an apatite layer upon immersion are carried out in “close-to-physiological” media. Generally, though, only the mineral fraction of body fluids is considered (e.g., in SBF solution [19]), meaning in particular in the absence of proteins or cells. This situation indeed allows a greater ease of use and leads already to some informative results. However, it should be kept in mind at this point that the adsorption of organic substances such as proteins or peptides, or the activity of cells once in actual *in vivo* environments, could lead to somewhat modified outcomes. 

**Figure 1:**
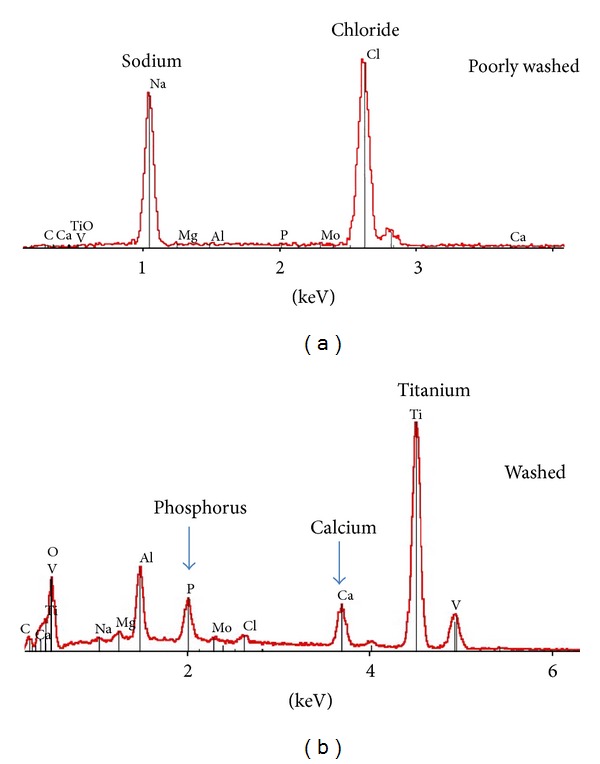
SEM and EDX observations for a TA6V disk immersed in SBF × 10 for 5 days at RT—role of washing.

In cases when synthetic simulated body fluids (only mineral) are prepared, it is customary to use chemical compositions that are more or less close to that of blood plasma (reported in Table 1, as reported by Krebs [25], along with speciation data determined in this work using the Visual Minteq 3.0 software). As can be seen, blood plasma (or synthetic SBF) contains in particular large amounts of sodium and chloride ions, which are predominant ionic species. The observation of a white deposit on the substrate after withdrawal of the piece is therefore not an absolute proof of the formation of apatite at this stage; indeed, in cases where the washing step has not been thoroughly performed, the formation/residual presence of NaCl aggregates could explain this white coating phenomenon.  Figure 1 reports the illustrative example of a titanium alloy disk (TA6V) immersed in SBF × 10 for 5 days at room temperature (RT) in the case of either incomplete or more advanced washing. Although SEM observations evidenced the presence of deposited crystals in both cases, EDX analyses pointed out that the poorly washed sample was (as one could indeed expect) mostly covered by NaCl crystals. In the context of immersion tests, the absence of NaCl crystals should then probably be systematically verified (by EDX and/or XRD analyses).

#### 2.1.2. Role of “Real” Experimental Conditions

In cases where adequate sample preparation was done prior to analysis, thus including thorough washing steps, the observation of a deposit after immersion in a hydroxyapatite-supersaturated medium still does not necessarily imply the formation of apatite. Experimental mistakes (e.g., due to the weighing of hygroscopic starting salts for preparing the supersaturated solution) may lead to undesired immersion conditions and then to nucleated phases other than apatite. This stresses the fact that parameters such as the medium pH should probably be monitored during the immersion step to verify that no significant pH change has occurred. This is all more relevant as the precipitation of (hydroxy) apatite implies the incorporation of OH^−^ ions and theoretically implies an acidification of the medium: this acidification phenomenon may remain of low amplitude if the liquid-to-solid ratio is chosen sufficiently high, but this underlines the fact that in some cases the pH may turn out to be inappropriate to form apatite. The incorporation of pH-buffering agents like Tris can be found effective, but this implies the addition to the medium of a “foreign” organic compound, which may not always appear appropriate. In cases when the pH follows uncontrolled variations, other phases may stably form instead of apatite (depending on the exact conditions), such as acidic calcium phosphate compounds like brushite CaHPO_4_·2H_2_O (DCPD), monetite CaHPO_4_ (DCPA) or octacalcium phosphate (OCP). Indeed, these phases become more stable than hydroxyapatite in acidic pH conditions [24]. 

The variation of supersaturation during immersion tests may also lead to a modification in deposited phase with time. For information, the saturation index relative to various phases of interest and considering as initial conditions the ionic concentrations of blood plasma (inorganic species, see Table 1) is reported in Table 2. A negative value of this index is indicative of a situation where the medium is supersaturated with respect to the phase concerned. These data were calculated using the Visual Minteq 3.0 software, taking into account the solubility products that are generally accepted for these various phases (added in Table 2). As may be noted, such conditions (if correctly set up from an experimental point of view) lead to a high supersaturation with respect to HA and to a moderate supersaturation for octacalcium phosphate (OCP), calcite or vaterite, among other phases. In contrast, they are moderately undersaturated with respect to magnesium-containing phases such as MgHPO_4_·3H_2_O and more undersaturated with respect to brushite or monetite. If undesirable (or variations in) ionic concentrations are present during the tests, however, the ionic product is going to evolve and the overall saturation scheme is expected to change. Supersaturation aspects are thus also of prime importance during apatite precipitation plans.

In this regard, the use of constant composition crystal growth (C3G) systems [30, 31], which keeps the pH, ionic strength, temperature, and relative concentrations constant, can be seen as an advantageous way to control all experimental parameters and derive relevant data on the ability of substrates to provoke the nucleation and growth of apatite.

Finally, especially in the case of long immersion tests run in supersaturated fluids or for precipitations in media containing organic molecules (glucose, proteins…), the development of microorganisms may become significant for rather long periods of treatment. In this case, bacterial activity may then contribute to modifying the initial medium in a significant way. In order to avoid such issues, the incorporation of additives such as sodium azide NaN_3_ in the medium remains an option. Antibacterial agents such as pen-strep or fungizones may also be included. 

#### 2.1.3. Potential Impact of Substrate Equilibration with the Medium

The nature and behavior of the substrate itself, once in solution, may also play a nonnegligible role in the outcome of immersion tests. For instance, substrates exposing ionic or functional molecular grafted groups (e.g., after specific treatments aiming to alter the surface roughness or intending to improve cellular activity) may endure a preliminary surface equilibration with the medium, potentially modifying the initially determined immersion conditions [13]. If the pH of the medium, or another relevant factor, is altered by the conditioning of the substrate upon contact with the medium, this could in turn lead to uncontrolled or undesirable test conditions. Preliminary immersion tests should thus probably be run for samples for which the inert character has not been clearly established, so as to guarantee the absence of noticeable change in the medium. In this view, pH monitoring could again be seen as relevant during the process.

#### 2.1.4. Incomplete Reactions

Hydroxyapatite is a mineral phase that gathers a large number of ions. As such, its precipitation—especially at moderate temperatures—is bound to remain a rather slow phenomenon [24, 32]. This can explain the low degree of crystallinity of precipitated apatites, which subsequently mature in solution (evolution towards stoichiometry) at a pace that depends on experimental conditions [13]. In this context, the transient formation of precursor phases (metastable but exhibiting a faster formation rate) is also possible despite conditions that are thermodynamically favorable to apatite formation, in line with Ostwald's rule [33].

Among possible precursors, amorphous calcium phosphate (ACP) and octacalcium phosphate (OCP, triclinic) are two potential candidates [29, 34] that have already been suggested as precursors of bone-apatite *in vivo* [35, 36]. After their formation, both compounds may then be hydrolyzed to apatite in a subsequent step. ACP, although amorphous, is not totally exempt from structuration; it involves building blocks based on Ca_9_(PO_4_)_6_·*n*H_2_O clusters (so-called Posner clusters) [37], which can undergo partial internal hydrolysis through the reaction *x*PO_4_^3−^ + *x*H_2_O → *x*HPO_4_^2−^ + *x*OH^−^ [38], leading to Ca_9_(PO_4_)_6−*x*_(HPO_4_)_*x*_(OH)_*x*_ and allowing in particular the filling by OH^−^ ions of what will become apatitic channels. The hydrolysis of OCP probably involves a rather different mechanism. Taking into account the similarities between the structures of OCP and HA (detailed below) [39], the possibility of undergoing the OCP-to-apatite transformation via a topotactic mechanism appears as a possible scheme [40–42]. In this case, the usual plate-like morphology of OCP is expected to be rather conserved upon transformation into apatite. This illustrates then the difficulty of assessing phase identification uniquely on the basis of morphological features (as discussed below).

This fact points again to the necessity to adequately characterize formed phases, as even conditions that are thermodynamically consistent with the formation of an apatite phase may (at least transiently) allow the formation and sometimes stabilization of precursor compounds.

### 2.2. On the Characterization of Apatite Compounds

The above statements have unveiled various possible causes for immersion tests failure or mistaken precipitation situations. They illustrate in particular the possibility of obtaining deposited crystals other than apatitic, due to uncontrolled parameters like pH, supersaturation or substrate reactivity. This emphasizes the need for appropriate physico-chemical characterization of the phase formed (either on the occasion of immersion tests or more generally when intending to precipitate apatite compounds in any situation), in order to confirm or not the formation of an apatite phase. Several distinctive features (structural, compositional…) can be identified by the way of complementary techniques so as to draw conclusive statements. In this context, the following section aims to review the fundamentals of calcium phosphate apatite characterization. Comparisons with other phases that may also be encountered in humid conditions have also been included in the discussion.

#### 2.2.1. Microscopy Observations—TEM, SEM-EDX Techniques

Microscopy observations are often carried out, for example, for assessing the eventual presence of a deposit on the surface of a substrate immersed in supersaturated fluids. This is also the way to determine the morphological features of a precipitated compound. 

The detection of different morphologies during the analysis of a single sample should not be considered as insignificant as it may be indicative of the simultaneous presence of several phases in the specimen. This may, for example, be noticed on the occasion of immersion tests for which the solution-to-solid ratio has been underestimated, leading to strong variations in supersaturation levels along the test.

**Figure 2:**
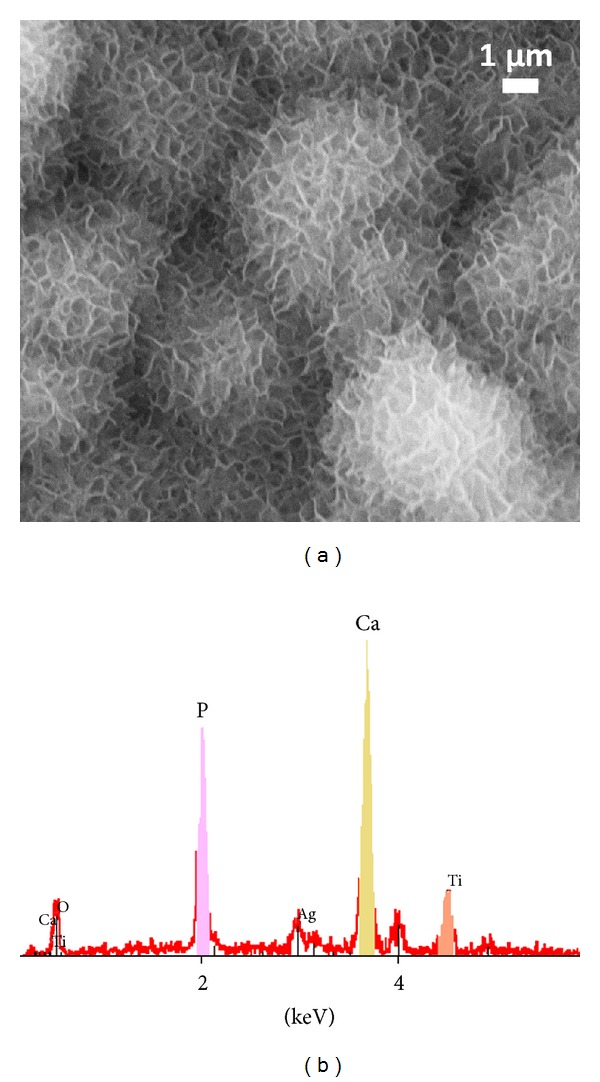
Usual plate-like morphology (SEM) for biomimetic apatite (example obtained after immersion of a titanium-based disk in Kim's supersaturated fluid [18] at 4°C for 4 days then 37°C for 4 days) and corresponding EDX spectrum.

In cases when a single morphology is observed (the best-case scenario), care should still be taken before drawing conclusions on the nature of the phase.  Figure 2 reports the usual plate-like or petal-like morphology of biomimetic apatite formed on the occasion of immersion tests in supersaturated solutions. Although the observation of this type of morphology is a promising factor in favor of the precipitation of bone-like apatite, this morphology should *not* be considered as an absolutely conclusive criterion. Indeed, other calcium phosphates may also exhibit anisotropic or plate-like morphologies. Platelets are also characteristic, for example, of monetite (CaHPO_4_) or brushite (CaHPO_4_·2H_2_O), although the particle size is generally larger than for bone-like nanocrystalline apatites. Triclinic OCP also exhibits a petal-like morphology. Moreover, unexpected remaining impurities (e.g., due to poor washing) may also lead to morphological similarities with biomimetic apatite.

EDX elemental analysis can be considered as a precious (although still not sufficient) tool for unveiling compositional aspects. It may, for example, rule out the presence of NaCl crystals (Figure 2). The observation of calcium and phosphorus lines is then in favor of the formation of a calcium phosphate phase. However, although quantitative Ca/P molar ratios may be drawn from EDX analyses (if appropriately compared with elemental standards), it should be remembered that bone-like apatites are often largely nonstoichiometric and exhibit a nonapatitic surface layer hosting mostly bivalent ions such as HPO_4_^2−^ instead of PO_4_^3−^ (which may be related to the conditions of formation of such apatites in solution) [2, 13, 43–48]. Therefore, the Ca/P ratio *cannot* be exploited to unequivocally determine the nature of the calcium phosphate phase (e.g., noncarbonated nanocrystalline apatites with a Ca/P ratio as low as 1.30 have been prepared [13])—this point will be addressed again in the “chemical analyses” subsection.

Consequently, while morphological and elemental EDX analyses are valuable for first characterization statements, they cannot be exclusively used to confirm the apatitic nature of the precipitated phase, and additional characterization is needed.

#### 2.2.2. X-Ray Diffraction (XRD)

**Figure 3:**
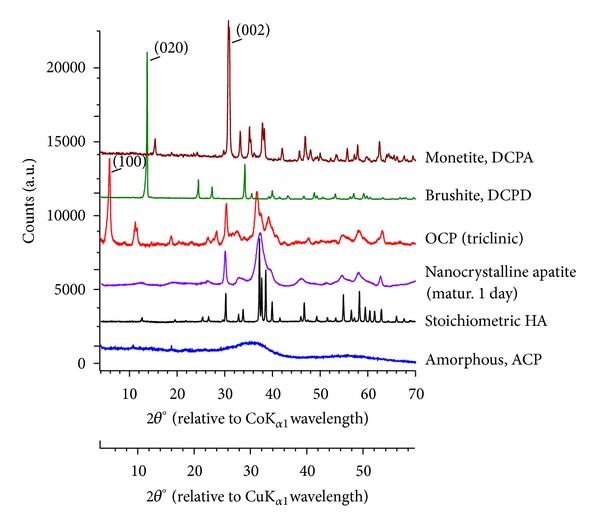
Characteristic XRD patterns for a well-crystallized stoichiometric hydroxyapatite (HA), a nanocrystalline apatite (matured 1 day), octacalcium phosphate (OCP), amorphous calcium phosphate (ACP), monetite (DCPA), and brushite (DCPD) (relative to either *λ*CoK_*a*1_ or *λ*CuK_*a*1_).

XRD is undeniably a central characterization tool for identifying precipitated crystallized phases. On the contrary, amorphous compounds (sometimes present beside a crystallized phase) will only lead to large halos, which are unfortunately sometimes overlooked. In the case of calcium phosphates, the halos are due to amorphous tricalcium phosphate (ACP) and arise as background deformations under the main diffraction lines of apatitic compounds (see Figure 3), corresponding to interreticular distances approximately in the ranges 1.60–2.26 and 2.30–4.07Å. Care should consequently be taken when inspecting these regions so as to verify the absence of broad halos. 

The XRD pattern of well-crystallized stoichiometric hydroxyapatite (HA) is also reported in Figure 3 (relative to both cobalt and copper anticathode K_*α*1_ wavelengths). In cases when acidic pH values may be reached, other calcium phosphate phases such as monetite or brushite may form, the patterns of which have also been added in Figure 3. At this point, it should also be remembered that the acquisition of XRD diagrams often favors preferential crystal orientations. This is especially true for phases exhibiting highly anisotropic morphologies like monetite or brushite. In such cases, experimental XRD patterns may then significantly depart from calculated ones, by variations in peak intensities. A noticeable modification of the XRD pattern is, for example, found for brushite, for which the (020) experimental line becomes noticeably more intense than expected for randomly oriented crystals. Despite such pattern modifications, the XRD profiles of monetite and brushite distinctively depart from that of apatite compounds, allowing one to rule out or not the presence of such phases.

Preferential orientations and related peak intensity modifications are also observable for precipitated OCP, but in this case the distinction from an apatite profile is more subtle (Figure 3). The relative similarity of experimental patterns obtained for OCP and a poorly-crystalline apatite may in fact be related to (partial) structural resemblances; the OCP structure can indeed be described as the alternative stacking of “apatitic” layers (with crystallographic positing of ions very close to those in HA) and “hydrated” layers (which enclose in particular all the HPO_4_^2−^ ions contained in OCP) [39]. In this context, the distinctive presence of the (100) line of OCP at very low 2*θ* angles (see Figure 3) then becomes particularly helpful for distinguishing, on the basis of XRD analyses, precipitated OCP from an apatite phase. Unfortunately, low angle ranges are not always examined or reported in the literature studies (the diffractometer setup must sometimes be specifically adapted to access this low-angle region).

XRD analysis is thus one major stage in sample characterization. But limitations to this technique also exist as illustrated above (difficulty in revealing the presence of amorphous compounds, existence of preferential orientations, and necessity to analyze specific angle ranges). Also, in the case of immersion tests, the deposit to be expected often remains of very limited depth, making XRD analyses more difficult to perform (the analysis of thin deposits being more effectively carried out with glazing angle analyses); complementary analysis may then be required, including in particular spectroscopy techniques that are addressed below.

#### 2.2.3. Vibrational Spectroscopies: FTIR and Raman

In view of the preceding statements, crystallographic data are often helpful but not always conclusive in terms of phase identification. Vibrational spectroscopies (FTIR, Raman) can then be seen as useful tools for gaining more insight on the constitutive ions of a sample and related ionic environments. Indeed, phosphate and hydroxide groups—which involve covalent bonds—lead to very specific vibrational features when involved in an apatitic system, which may be exploited for phase identifications.

**Figure 4:**
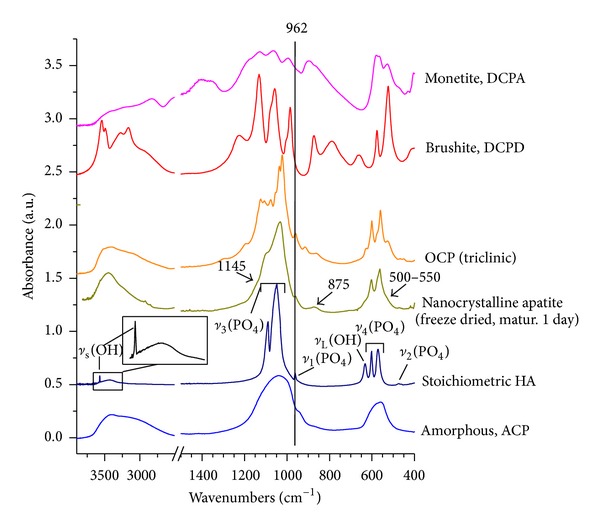
Characteristic FTIR spectra (400–1500 cm^−1^ range) for a well-crystallized stoichiometric hydroxyapatite (HA), a nanocrystalline apatite (matured 1 day, freeze dried), octacalcium phosphate (OCP), amorphous calcium phosphate (ACP), monetite (DCPA), and brushite (DCPD).

Table 3 reports the typical vibrational features of PO_4_^3−^ and OH^−^ ions in well-crystallized stoichiometric HA, and Figure 4 reports characteristic FTIR spectra for various calcium phosphates of interest in this study (obtainable in wet conditions), namely, stoichiometric HA, a nanocrystalline apatite matured for 1 day (freeze dried), OCP, ACP, monetite, and brushite. This figure may be used to identify some major spectral differences among these phases. In the case of HA, two bands due to OH^−^ ions in apatitic environments are in particular detectable at 632 ± 2 (as a shoulder to the *ν*_4_(PO_4_) band) and 3572 ± 2 cm^−1^ (narrow peak). However, for nonstoichiometric poorly-crystallized apatites obtained by precipitation at moderate temperatures, these OH bands are often difficult to detect by IR spectroscopy (see Figure 4); the low degree of crystallinity tends to enlarge vibrational bands, covering weak OH signals, and the nonstoichiometry disfavors the presence of OH^−^ ions in apatitic channels. Also, such precipitated apatites are generally associated with water molecules, leading to strong absorption in the 3000–3700 cm^−1^ region. The peak at 3572 cm^−1^ is, however, also active in Raman spectroscopy, where water molecules are not significantly perceptible. The detection of this band (not to be confused with the strong absorption of brushite in this region, Figure 4) is one factor attesting to the presence of an apatite phase. 

Besides, although the *ν*_1_(PO_4_) band at 962 ± 2 cm^−1^ is only of minor intensity in IR spectroscopy (Table 3), its presence is also of interest. It is indeed observed at this position in apatite phases or OCP but not in ACP (band shifted to lower wavenumbers, leading to a shoulder to the *ν*_3_(PO_4_) band, around 950 cm^−1^), monetite (band shifted to ca. 995 cm^−1^), nor brushite (band shifted to ca. 985 cm^−1^), as shown in Figure 4. Besides, this *ν*_1_(PO_4_) band is very active in Raman spectroscopy, which may allow further identification validation.

FTIR spectra of nanocrystalline (biomimetic) apatites show modified features compared with well-crystallized stoichiometric HA. First of all, spectra are often composed of enlarged bands due to rather low degrees of crystallinity (Figure 4). In these conditions, bands that appeared isolated in HA may only give rise to merged groups of bands (the decomposition of which requires mathematical treatment). Moreover, as mentioned above, nanocrystalline apatites are generally nonstoichiometric—with calcium and hydroxide vacancies—, and the limited amount of OH^−^ ions in apatitic channels leads to added difficulty in detecting such ions. However, in addition to the “regular” apatite spectral features observed in HA, nanocrystalline apatites also exhibit supplementary bands which are due to the presence of nonapatitic ionic environments within a surface layer on the nanocrystals, as mentioned in the introduction section; bands due to nonapatitic (labile) HPO_4_^2−^ and PO_4_^3−^ as well as CO_3_^2−^ ions have in particular been identified [7, 13, 46, 47, 49–52], and spectral decompositions are then needed to explore in further details each vibrational contribution (see e.g., [13]). Also, in addition to nonapatitic HPO_4_^2−^ ions, some apatitic HPO_4_^2−^ ions are generally present in the lattice. 

From a “practical” viewpoint, some modifications of IR spectra can be identified in the case of nanocrystalline biomimetic apatites—as compared to HA—due to the presence of HPO_4_^2−^ ions associated with the apatite crystals, as indicated by arrows in Figure 4. This includes (1) a broad shoulder on the low wavenumbers region of the *ν*_4_(PO_4_) band (shoulder typically expanding from 500 to 550 cm^−1^ that in fact comprises two subcomponents centered around 530–534 and 550 cm^−1^ assignable, respectively, to nonapatitic and apatitic HPO_4_^2−^) and (2) a shoulder on the high wavenumbers region of the *ν*_3_(PO_4_) band (visible on spectra by a burst around 1145 cm^−1^). A thin band at 875 cm^−1^ is also detected for HPO_4_-bearing apatites due to the P-OH stretching. However, if the apatite is partly carbonated (which is noticeable for instance from a broad *ν*_3_(CO_3_) absorption band in the region 1350–1570 cm^−1^), then the HPO_4_ band at 875 cm^−1^ becomes almost superimposed with the *ν*_2_(CO_3_) band (around 872 cm^−1^ for B-type carbonate ions), making it difficult to exploit. 

**Figure 5:**
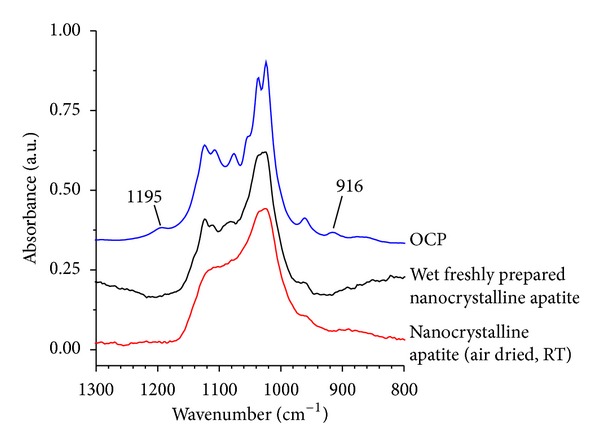
Comparison of FTIR features for nanocrystalline apatite (dried and undried) and OCP in the *ν*_3_(PO_4_) vibration range 800–1300 cm^−1^.

The above statements indicate that several parameters may allow one to distinguish apatites from other calcium phosphates. Similarities, however, do remain between the spectral features of nanocrystalline apatites and precipitated OCP, which can be attributed to the above-mentioned structural relationship. Such similarities are even more obvious when analyzing fresh precipitates; indeed, in these conditions the nonapatitic layer on the surface of the nanocrystals is likely to remain rather intact (i.e., not altered by drying processes), which gives rise to a better-defined IR spectrum than for dried specimens (Figure 5). However, a closer look at the spectra, especially in the *ν*_3_(PO_4_) region, indicates that two distinct bands at ca. 1195 and 916 cm^−1^ (assignable to HPO_4_^2−^ ions in the OCP lattice configuration) are detectable for OCP but not for apatite. These differences may then be considered as other elements for distinguishing between these two phases by IR spectroscopy.

Finally, additional identification information can come from the possible presence of carbonate vibration bands, the position of which has been cited above. Indeed, while the apatite structure can accommodate carbonate CO_3_^2−^ ions, this is not an option for OCP. The detection of such carbonate bands can then be taken as an additional confirmation of apatite formation.

Vibrational spectroscopies, and in particular FTIR, thus represent particularly well-suited tools for phase identifications in the Ca-PO_4_-H_2_O system, especially for confirming or not the “apatite” nature of a given sample.

#### 2.2.4. Chemical Analyses

The detection of calcium and phosphorus by EDX analyses has been mentioned previously. The oxidation state of phosphorus in orthophosphate ions used in starting salts or in phosphoric acid is +V; since highly reductive conditions are generally not found for the type of experiments of interest in this paper, this oxidation state is bound to remain unchanged; the observation of phosphorus by EDX may be considered in such cases as a mark of the presence of phosphate ions. The titration of phosphate ions can, however, be performed more accurately using a chemical method, for example, via visible spectrophotometry (using the phosphovanadomolybdenum complex formed in acidic conditions) [53, 54]. Other techniques may also be used to this end, such as ICP-AES, but our experience shows that levels of phosphate ions obtained by this technique often tend to be underestimated and the analysis of a good reference sample such as calcined stoichiometric HA should probably always be concomitantly done for cross-checking and potentially applying data correction.

The calcium content of the formed compound can be reached without significant issues, for example, either by complexometry (e.g., with EDTA), via atomic absorption, or ICP techniques. If XRD, SEM, and FTIR data converge to show that the sample is single phased and apatitic in nature, then these titrations may allow the Ca/P molar ratio of the specimen to be determined. However, the apatite structure is well known for its ability to host a great number of different substituents that are also often present in precipitating or immersion media such as sodium (Na^+^) or magnesium (Mg^2+^) ions. Thus, the Ca/P ratio may not be the best parameter to evaluate the overall nonstoichiometry of the apatite phase. Indeed, if other cations are also present in the structure, then the “cations/P” ratio should be evaluated instead. And this also holds if anionic species are incorporated in phosphate sites (such as B-type carbonate ions whose presence may be detected by FTIR analyses, as mentioned above). Therefore, apatite's overall nonstoichiometry should be evaluated from a more general consideration of cations in calcium sites over anions in phosphate sites, which I propose to denote as Ca*/P*. This nonstoichiometry parameter should, however, only be taken as an informative datum, since (as we mentioned previously) nanocrystalline apatites may exhibit Ca*/P* ratios in a wide range, typically from ca. 1.30 up to 1.67.

Therefore, chemical titrations appear as an interesting additional tool for the detailed characterization of the apatite phase obtained, but its apatitic nature should be previously confirmed by other techniques, as illustrated above (XRD, SEM, vibrational spectroscopy, and/or solid state NMR as is discussed below).

#### 2.2.5. Solid State NMR

Although it is not always available on site, solid state NMR remains another helpful technique for the exploration of physico-chemical characteristics of calcium phosphate phases. ^1^H and ^31^P MAS-NMR spectra were found to be particularly attractive for gaining information on the local chemical environment of phosphates ions in such compounds (see, e.g., [48, 55, 56]).

**Figure 6:**
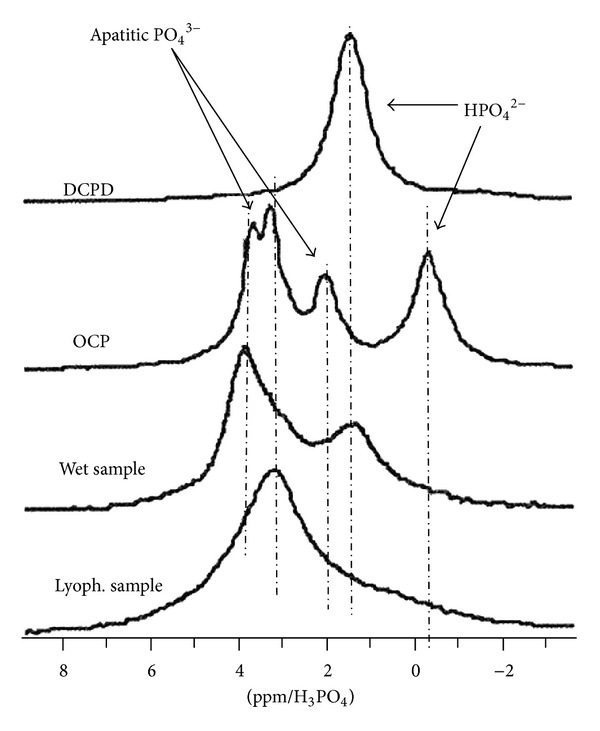
Solid state ^31^P MAS-NMR spectra characteristic of nanocrystalline apatites (wet or lyophilized), OCP, and DCPD (reprinted with permission from [43]).

In the case of stoichiometric HA, ^31^P MAS-NMR data show a unique peak around 2.8 ppm (with respect to H_3_PO_4_), which corresponds to apatitic PO_4_^3−^ ions [57]. In contrast, for nanocrystalline apatites, additional contributions were observed, taking the form of a dissymmetric peak with a maximum (for dried samples) around 3.1 ppm (wet samples exhibit a peak maximum at greater fields, around 3.7 ppm [55]) and a shoulder at 1.4 ppm, which was related to HPO_4_^2−^ ions associated with the apatite phase (Figure 6). The position of this shoulder is indeed similar to that of HPO_4_^2−^ ions in brushite [43]. However, in contrast to FTIR data for which some similarities were found between nanocrystalline apatite and OCP (see FTIR subsection above), it may be noted that there are no direct similarities between the NMR characteristics of OCP and apatite. Indeed, OCP is instead characterized by a very distinct ^31^P profile, with peaks at −0.3, 2, 3.2, and 3.5 ppm [43]. These significant differences can therefore be exploited for phase identification purposes. 

Finally, proton ^1^H NMR spectra can also be useful for apatite phase identification due to the presence of a characteristic peak at 0.1-0.2 ppm (relative to TMS) attributable to apatitic OH^−^ ions [48, 55, 58].

The solid state NMR technique therefore appears as another interesting tool, especially for distinguishing between OCP and apatite.

## 3. Experimental Section

### 3.1. Compounds Synthesis

All reagents used in this study were reagent grade. 

Stoichiometric hydroxyapatite (HA), Ca_10_(PO_4_)_6_(OH)_2_, was prepared by adding dropwise and underreflux a solution of diammonium hydrogenphosphate into a boiling solution of calcium nitrate, in stoichiometric proportions and in ammoniated conditions. Note that for this preparation, decarbonated water (previously boiled up) was used throughout the process. After 4 hours (at pH~10), the precipitate was filtered, thoroughly washed with deionized water, and dried at 100°C prior to calcination at 1000°C for 1 h. 

The nanocrystalline apatite samples studied in this work were prepared by precipitation from mixing aqueous solutions of diammonium hydrogenphosphate in excess (0.6 M) and calcium nitrate (0.3 M), at 22°C and at pH = 7.2 close to the physiological value. The excess of phosphate ions was used to provide an internal pH buffer without any addition of external buffer agent. After rapid mixing (1 min), the precipitates were left to mature in solution for 1 day and then filtered, washed, and freeze dried.

Brushite, CaHPO_4_·2H_2_O, was synthesized via precipitation by stoichiometric addition of an ammoniated calcium nitrate solution into a solution of ammonium dihydrogenphosphate, followed by a 2-hour maturation stage. The precipitate was then filtered, washed, and dried at 37°C overnight.

Monetite, CaHPO_4_, was obtained by heat treatment of brushite (at 180°C) in air.

Amorphous calcium phosphate (ACP), am-Ca_3_(PO_4_)_2_, was prepared by rapidly adding an ammoniated solution of calcium nitrate into an ammoniated solution of diammonium hydrogenphosphate, followed by a direct filtration, washing with ammoniated water, and freeze drying for 24 hours.

Octacalcium phosphates (OCP, triclinic), Ca_8_(PO_4_)_4_(HPO_4_)_2_·5H_2_O, was obtained by hydrolysis, under stirring, of brushite crystals in the presence of an identical weight of ammonium hydrogenphosphate, at 37°C for 24 hours. This was followed by a step of filtration, washing, and freeze drying. The sample was then conserved at low temperature (−18°C) to avoid any evolution.

When appropriate (mentioned in the text), simulated body fluid (SBF) and 10-times concentrated SBF (10 × SBF) were prepared by following Kokubo's protocol [19] or by multiplying all concentrations by a factor of 10, respectively. 

### 3.2. Physicochemical Characterization

X-ray diffraction (XRD) analyses were carried out on an INEL 120 CPS curved counter diffractometer using the CoK_*α*1_ radiation (with *λ* = 1.78892 Å). 

Fourier transform infrared (FTIR) spectra were obtained on a Nicolet 5700 spectrometer, using the KBr pellet method, in the range 400–4000 cm^−1^ (64 scans, resolution 4 cm^−1^).

SEM-EDX analyses were performed on a LEO 435 VP microscope operated at 10–15 kV.

## 4. Conclusions

This review was aimed at remembering the necessity and the means to carry out adequate physico-chemical characterization prior to drawing conclusive results on the apatitic nature of a given sample or deposit. 

Several possible reasons for experimental failure were brought up in the first part of this paper, where various aspects that are not always taken into account were underlined (possible experimental mistakes, poor washing step, evolution of a substrate in solution, contamination of a medium by microorganisms, residual presence of precursor phases, etc.). 

The second part of this review considered the fundamentals of apatite characterization based on various complementary techniques. Even in thermodynamically adequate conditions, some kinetic aspects or other factors—such as the presence of a stabilizing impurity in the medium—could mislead the experimentalist and give rise to unexpected results. In the case of immersion tests in supersaturated solutions, for example, the formation of a white deposit, even characterized by a plate-like morphology, does not necessarily indicate the formation of apatite. Characterization tools like XRD, FTIR/Raman, or solid state NMR prove to be especially well suited to reliably identify apatite compounds, including distinguishing between OCP and nanocrystalline apatite.

All that glitters is not gold… all that is white is not apatite either.

## Figures and Tables

**Table 1:** Chemical composition of blood plasma (inorganic species) from Krebs [25], at pH 7.4 and 37°C, and speciation data (calculated using the Visual Minteq 3.0 software).

Main ion types	Average global concentration (mM)	Main species (and % of total concentration*)	Corresponding activities** (mM)
Sodium	137.4	Na^+^ (97%)	**100.900**

Chloride	103	Cl^−^ (96%)	**75.427**

Carbonate	27	HCO_3_^−^ (90%)	**18.509**
		H_2_CO_3(aq)_ (5%)	1.486

Potassium	4.4	K^+^ (97%)	**3.246**

Calcium	2.5	Ca^2+^ (79%)	**0.655**
		CaHCO_3_^+^ (9%)	0.177
		CaCl^+^ (7%)	0.132

Phosphate (inorganic)	1	HPO_4_^2−^ (45%)	**0.151**
		NaHPO_4_^−^ (30%)	0.226
		H_2_PO_4_^−^ (12%)	0.090
		CaHPO_4(aq)_ (5%)	0.054

Magnesium	0.8	Mg^2+^ (78%)	**0.207**
		MgCl^+^ (11%)	0.066
		MgHCO_3_^+^ (7%)	0.042

Sulfate (inorganic)	0.5	SO_4_^2−^ (75%)	**0.125**
		NaSO_4_^−^ (19%)	0.071

*For species in amount ≥5%.

**Using Davies' equation for calculation of activity coefficients (total ionic strength ~0.14 M).

**Table 2:** Saturation index relative to various phases of interest and considering as initial conditions the ionic concentrations of blood plasma reported in Table 1.

Selected phases	*pK*_sp_ (25°C)	Reference	Saturation index^(c)^ for blood plasma conditions (kJ/mol)
Hydroxyapatite (HA) Ca_10_(PO_4_)_6_(OH)_2_	116.8^(a)^	[26]	−**15.673**
Octacalcium phosphate (OCP) Ca_8_(PO_4_)_4_(HPO_4_)_2_·5H_2_O	96.6^(b)^		−**1.540**
Monetite (DCPA) CaHPO_4_	6.9		24.875
Brushite (DCPD) CaHPO_4_·2H_2_O	6.59		25.381

Calcite c-CaCO_3_	8.48	[27]	−**2.377**
Vaterite v-CaCO_3_	7.91		−**0.788**

Magnesium hydrogenphosphate MgHPO_4_·3H_2_O	18.18	[28]	3.171

^(a)^Expressed relative to HA formula in Ca_10_.

^(b)^Expressed relative to OCP formula in Ca_8_.

^(c)^Saturation index (Δ*G*_*s*_) [29] calculated as: Δ*G*_*s*_ = −(*RT*/*N*)Ln(*IP*/*K*_sp_) where *N* is the number of ions involved, IP is the ion product, and *K*_sp_ is the solubility product. A negative value of Δ*G*_*s*_ means that the medium is supersaturated with respect to the concerned phase.

**Table 3:** FTIR characteristic bands (PO_4_^3−^ and OH^−^) for stoichiometric HA.

Hydroxyapatite (HA) Ca_10_(PO_4_)_6_(OH)_2_		
Band position (cm^−1^) (±2 cm^−1^)	Relative intensity	Attributions
PO_4_^3−^ groups (apatitic)		
474 (isolated)	very weak	*ν*_ 2_(PO_4_): OPO bending
562 and 575 (merged together)	moderate	*ν*_ 4_(PO_4_): OPO bending
603 (isolated)	moderate	*ν*_ 4_(PO_4_): OPO bending
**962** (isolated)	weak	*ν*_1_(PO_4_): PO stretching
1048, 1090 (both isolated)	intense	*ν*_ 3_(PO_4_): PO stretching

OH^−^ groups (apatitic)		
**632** (shouldered to *ν*_4_(PO_4_))	moderate/weak	*ν*_*L*_(OH): OH libration
**3572** (shouldered to water O–H)	moderate/weak	*ν*_*s*_(OH): OH stretching

## References

[1] Gomez-Morales J, Iafisco M, Delgado-Lopez JM, Sarda S, Drouet C (2013). Progress on the preparation of nanocrystalline apatites and surface characterization: overview of fundamental and applied aspects. *Progress in Crystal Growth and Characterization of Materials*.

[2] Cazalbou S, Combes C, Eichert D, Rey C, Glimcher MJ (2004). Poorly crystalline apatites: evolution and maturation in vitro and in vivo. *Journal of Bone and Mineral Metabolism*.

[3] Grynpas M (1976). The crystallinity of bone mineral. *Journal of Materials Science*.

[4] Handschin RG, Stern WB (1992). Crystallographic lattice refinement of human bone. *Calcified Tissue International*.

[5] Handschin RG, Stern WB (1995). X-ray diffraction studies on the lattice perfection of human bone apatite (Crista iliaca). *Bone*.

[6] Johansen E, Parks HF (1960). Electron microscopic observations on the three-dimensional morphology of apatite crystallites of human dentine and bone. *The Journal of Biophysical and Biochemical Cytology*.

[7] Rey C, Lian J, Grynpas M, Shapiro F, Zylberberg L, Glimcher MJ (1989). Non-apatitic environments in bone mineral: FT-IR detection, biological properties and changes in several disease states. *Connective Tissue Research*.

[8] Rey C, Combes C, Drouet C, Glimcher MJ (2009). Bone mineral: update on chemical composition and structure. *Osteoporosis International*.

[9] Drouet C, Carayon M, Combes C, Rey C (2008). Surface enrichment of biomimetic apatites with biologically-active ions Mg2+ and Sr2+: a preamble to the activation of bone repair materials. *Materials Science and Engineering C*.

[10] Drouet C, Gómez-Morales J, Iafisco M, Sarda S, Rimondini L, Bianchi CL, Vernè E (2012). Calcium phosphate surface tailoring technologies for drug delivering and tissue engineering and applied aspects. *E-Book: Surface Tailoring of Inorganic Materials for Biomedical Applications*.

[11] Winand L (1961). Etude physic-chimique du phosphate tricalcique hydrate et de l’hydroxyapatite. *Annali di Chimica*.

[12] Kühl G, Nebergall WH (1963). Hydrogenphosphat- und carbonatapatite. *Zeitschrift für Anorganische und Allgemeine Chemie*.

[13] Vandecandelaere N, Rey C, Drouet C (2012). Biomimetic apatite-based biomaterials: on the critical impact of synthesis and post-synthesis parameters. *Journal of Materials Science-Materials in Medicine*.

[14] Hallab N, Merritt K, Jacobs JJ (2001). Metal sensitivity in patients with orthopaedic implants. *Journal of Bone and Joint Surgery A*.

[15] Albrektsson T, Branemark PI, Hansson HA (1983). The interface zone of inorganic implants in vivo: titanium implants in bone. *Annals of Biomedical Engineering*.

[16] Bidar R, Kouyoumdjian P, Munini E, Asencio G (2009). Long-term results of the ABG-1 hydroxyapatite coated total hip arthroplasty: analysis of 111 cases with a minimum follow-up of 10 years. *Orthopaedics and Traumatology*.

[17] De Groot K, Geesink R, Klein CPAT, Serekian P (1987). Plasma sprayed coatings of hydroxylapatite. *Journal of Biomedical Materials Research*.

[18] Kim H, Kim Y, Park S (2000). Thin film of low-crystalline calcium phosphate apatite formed at low temperature. *Biomaterials*.

[19] Oyane A, Kim H, Furuya T, Kokubo T, Miyazaki T, Nakamura T (2003). Preparation and assessment of revised simulated body fluids. *Journal of Biomedical Materials Research A*.

[20] Ratner BD, Hoffman AS, Schoen FJ, Lemons JE (1996). *Biomaterials Science: An Introduction to Materials in Medicine*.

[21] Abe Y, Kokubo T, Yamamuro T (1990). Apatite coating on ceramics, metals and polymers utilizing a biological process. *Journal of Materials Science*.

[22] Jalota S, Tas AC, Bhaduri SB In vitro comparison of the apatite inducing ability of three different SBF solutions on Ti6Al4V.

[23] Tas AC (2000). Synthesis of biomimetic Ca-hydroxyapatite powders at 37°C in synthetic body fluids. *Biomaterials*.

[24] Elliott JC (1994). *Structure and Chemistry of the Apatites and Other Calcium Orthophosphates*.

[25] Krebs HA (1950). Chemical composition of blood plasma and serum. *Annual Review of Biochemistry*.

[26] Tung MS, Amjad Z (1998). Calcium phosphates: structure, composition, solubility, and stability. *Calcium Phosphates in Biological and Industrial Systems*.

[27] Plummer LN, Busenberg E (1982). The solubilities of calcite, aragonite and vaterite in CO_2_-H_2_O solutions between 0 and 90°C, and an evaluation of the aqueous model for the system CaCO_3_-CO_2_-H2O. *Geochimica et Cosmochimica Acta*.

[28] Smith RM, Martell AE, Motekaitis RJ (2003). *NIST Crititically Selected Stability Constants of Metal Complexes Database*.

[29] Eanes ED, Meyer JL (1977). The maturation of crystalline calcium phosphates in aqueous suspensions at physiologic pH. *Calcified Tissue International*.

[30] Spanos N, Misirlis DY, Kanellopoulou DG, Koutsoukos PG (2006). Seeded growth of hydroxyapatite in simulated body fluid. *Journal of Materials Science*.

[31] Tomson MB, Nancollas GH (1978). Mineralization kinetics: a constant composition approach. *Science*.

[32] Nancollas GH, Tomažič B (1974). Growth of calcium phosphate on hydroxyapatite crystals. Effect of supersaturation and ionic medium. *Journal of Physical Chemistry*.

[33] Feenstra TP, De Bruyn PL (1981). The ostwald rule of stages in precipitation from highly supersaturated solutions: a model and its application to the formation of the nonstoichiometric amorphous calcium phosphate precursor phase. *Journal of Colloid And Interface Science*.

[34] Meyer JL, Eanes ED (1978). A thermodynamic analysis of the amorphous to crystalline calcium phosphate transformation. *Calcified Tissue International*.

[35] Mahamid J, Sharir A, Addadi L, Weiner S (2008). Amorphous calcium phosphate is a major component of the forming fin bones of zebrafish: indications for an amorphous precursor phase. *Proceedings of the National Academy of Sciences of the United States of America*.

[36] Brown WE, Lehr JR, Smith JP, William Frazier A (1957). Crystallography of octacalcium phosphate. *Journal of the American Chemical Society*.

[37] Posner AS, Betts F (1975). Synthetic amorphous calcium phosphate and its relation to bone mineral structure. *Accounts of Chemical Research*.

[38] Heughebaert JC (1977). *Contribution à l'Étude de l'Évolution des Orthophosphates de Calcium Précipités en Orthophosphates Apatitiques*.

[39] Brown WE, Schroeder LW, Ferris JS (1979). Interlayering of crystalline octacalcium phosphate and hydroxylapatite. *Journal of Physical Chemistry*.

[40] Christoffersen J, Christoffersen MR, Kibalczyc W, Andersen FA (1989). A contribution to the understanding of the formation of calcium phosphates. *Journal of Crystal Growth*.

[41] Brown WE (1966). Crystal growth of bone mineral. *Clinical Orthopaedics and Related Research*.

[42] Tung MS, Brown WE (1983). An intermediate state in hydrolysis of amorphous calcium phosphate. *Calcified Tissue International*.

[43] Eichert D, Sfihi H, Combes C, Rey C (2004). Specific characteristics of wet nanocrystalline apatites. Consequences on blomaterials and bone tissue. *Key Engineering Materials*.

[44] Eichert D, Salomé M, Banu M, Susini J, Rey C (2005). Preliminary characterization of calcium chemical environment in apatitic and non-apatitic calcium phosphates of biological interest by X-ray absorption spectroscopy. *Spectrochimica Acta B*.

[45] Rey C, Combes C, Drouet C, Lebugle A, Sfihi H, Barroug A (2007). Nanocrystalline apatites in biological systems: characterisation, structure and properties. *Materialwissenschaft und Werkstofftechnik*.

[46] Rey C, Combes C, Drouet C, Sfihi H, Barroug A (2007). Physico-chemical properties of nanocrystalline apatites: implications for biominerals and biomaterials. *Materials Science and Engineering C*.

[47] Rey C, Combes C, Drouet C, Glimcher MJ (2009). Bone mineral: update on chemical composition and structure. *Osteoporosis International*.

[48] Jäger C, Welzel T, Meyer-Zaika W, Epple M (2006). A solid-state NMR investigation of the structure of nanocrystalline hydroxyapatite. *Magnetic Resonance in Chemistry*.

[49] Rey C, Collins B, Goehl T, Dickson IR, Glimcher MJ (1989). The carbonate environment in bone mineral: a resolution-enhanced Fourier Transform Infrared Spectroscopy study. *Calcified Tissue International*.

[50] Rey C, Shimizu M, Collins B, Glimcher MJ (1990). Resolution enhanced Fourier transform infrared spectroscopy study of the environment of phosphates ions in the early deposits of a solid phase of calcium phosphate in bone and enamel, and their evolution with age.1. Investigations in the *ν*4 domain. *Calcified Tissue International*.

[51] Rey C, Renugopalakrishnan V, Shimizu M, Collins B, Glimcher MJ (1991). A resolution-enhanced fourier transform infrared spectroscopic study of the environment of the CO32- ion in the mineral phase of enamel during its formation and maturation. *Calcified Tissue International*.

[52] Rey C, Renugopalakrishnan V, Collins B, Glimcher MJ (1991). Fourier transform infrared spectroscopic study of the carbonate ions in bone mineral during aging. *Calcified Tissue International*.

[53] Charlot G (1974). *Chimie Analytique Quantitative*.

[54] Eichert D, Drouet C, Sfihi H, Rey C, Combes C, Kendall JB (2008). Nanocrystalline apatite based biomaterials: synthesis, processing and characterization. *Biomaterials Research Advances*.

[55] Eichert D (2001). *Étude de la Reactivité de Surface d'Apatites de Synthèse Nanocristallines*.

[56] Wu Y, Glimcher MJ, Rey C, Ackerman JL (1994). A unique protonated phosphate group in bone mineral not present in synthetic calcium phosphates. Identification by phosphorus-31 solid state NMR spectroscopy. *Journal of Molecular Biology*.

[57] Tropp J, Blumenthal NC, Waugh JS (1983). Phosphorus NMR study of solid amorphous calcium phosphate. *Journal of the American Chemical Society*.

[58] Yesinowski JP, Eckert H (1987). Hydrogen environments in calcium phosphates: 1H MAS NMR at high spinning speeds. *Journal of the American Chemical Society*.

